# A Sexual Health Promotion App for Transgender Women (Trans Women Connected): Development and Usability Study

**DOI:** 10.2196/15888

**Published:** 2020-05-12

**Authors:** Christina J Sun, Kirsten M Anderson, Tamara Kuhn, Liat Mayer, Charles H Klein

**Affiliations:** 1 School of Public Health Oregon Health & Science University-Portland State University Portland, OR United States; 2 dfusion Scotts Valley, CA United States; 3 Department of Anthropology Portland State University Portland, OR United States

**Keywords:** transgender persons, HIV, sexual health, minority health, health care disparities, health status disparities

## Abstract

**Background:**

HIV severely impacts the transgender communities in the United States, and transgender women have the highest HIV incidence rates among any identified risk group. Guided by formative research with transgender women and by an expert advisory panel of transgender women, we designed a prototype mobile app to promote HIV prevention among transgender women.

**Objective:**

This study aimed to develop and test the usability and acceptability of the prototype Trans Women Connected mobile app.

**Methods:**

We engaged in a 3-phase prototype development process. After conducting formative research about the health needs of this population, we outlined a theory-based app framework and developed three prototype activities (ie, a vision board, a pre-exposure prophylaxis [PrEP] education activity, and an interactive map). We then tested the usability and acceptability of the mobile app and activities with 16 transgender women using pre- and posttests, think-aloud protocols, and open-ended questions.

**Results:**

Participants reported high acceptability for the mobile app; the mean rating across all usability and likability questions was 5.9 out of 7. Service utilization intention, goal setting, and social support increased at posttest compared with pretest. Increases in self-efficacy in finding lesbian, gay, bisexual, transgender, and queer–friendly services; intention to seek online social support; and PrEP knowledge were statistically significant. Participants described the app as attractive and useful and perceived all three activities positively.

**Conclusions:**

This study describes the development and usability and acceptability evaluation of a prototype mobile app designed for and with transgender women for HIV prevention. The usability testing findings provided important insights toward refining and the further development of the Trans Women Connected mobile app. The results suggest that a mobile health intervention can support positive changes. The remaining development and efficacy randomized trial of the Trans Women Connected mobile app is currently underway.

## Introduction

According to a 2016 meta-analysis, nearly 1 million adults in the United States are estimated to be transgender [1]. Transgender (or trans, in short) is a term for individuals whose gender expression and/or gender identity do not align with cultural expectations and gender norms associated with their sex assignment at birth [2]. Transgender women in the United States have been severely impacted by HIV [3-8], and more than 25% of them are living with HIV [5]. Transgender women of color are particularly affected; 56% of black/African American transgender women are living with HIV [5]. HIV incidence rates among transgender people are much higher than the national average [9] and highest among any group specifically tracked by the Centers for Disease Control and Prevention (CDC) [10]. Currently, the only transgender-specific program in the CDC’s Effective Interventions and Compendium of Evidence-Based Interventions and Best Practices for HIV [11,12] is an in-person, couples-based intervention designed for transgender women coupled with a cisgender male partner [13].

Internet/electronic health (eHealth) and mobile health (mHealth) modalities may be particularly appropriate for transgender women, many of whom are socially marginalized, who live in areas that do not offer transgender-specific programming and/or may be uncomfortable participating in face-to-face interventions because of confidentiality concerns and fear of stigmatization [5,14-16]. Although empirical data on internet and mobile app use among transgender persons is limited, existing studies show that transgender women consistently use their phones for information gathering, socializing, and making sexual connections [17-19]. These communication practices provide opportunities for engaging transgender women in HIV prevention and sexual health promotion issues. Mobile phone apps also have the capacity to deliver individually tailored content based on motivations for use and app use patterns [20], making them particularly appropriate for addressing the heterogeneous experiences, identities, and needs of transgender women across the life course. Furthermore, although there are very few studies evaluating transgender-specific eHealth or mHealth programs [21], a growing body of research about diverse communities shows that new media intervention programs are acceptable and can be effective in reducing HIV/sexually transmitted infection–related risk behaviors and linking individuals to prevention and care services [22-34]. Given these realities, program developers and practitioners are increasingly calling for transgender-specific internet-, social media-, and mobile-based programs to expand the reach of HIV prevention and health promotion activities [3,35-38].

The purpose of this study was to develop and evaluate the usability and acceptability of a prototype of the Trans Women Connected mobile app that addresses the unique HIV prevention and sexual health needs of transgender women. The goal was to engage transgender women through a strengths-based approach to HIV prevention and sexual health promotion, leveraging the power of social networks to identify and encourage protective factors.

## Methods

### Overview

**Figure 1:**
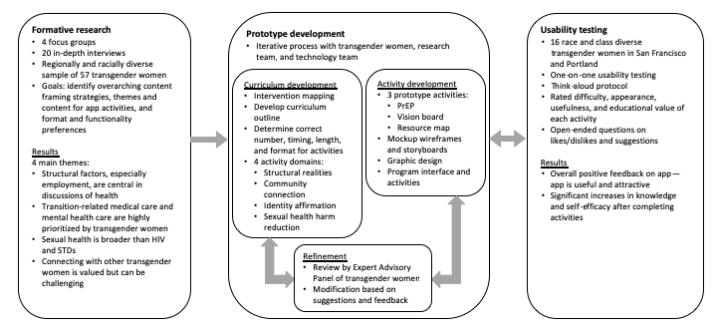
Prototype development process. PrEP: pre-exposure prophylaxis; STD: sexually transmitted disease.

We developed the prototype in three phases (see Figure 1). This process occurred in collaboration with a research team, an expert advisory panel (EAP), and a technology team. The EAP consisted of 3 transgender women of color, a Latinx woman, a Filipina woman, and an African American woman, each of whom holds a high-level position in an organization providing services to transgender women. They provided consultation and feedback throughout the prototype design and development process and were compensated as project consultants. The technology team included a technology director, an app developer, a database engineer, and a user experience and graphic designer and was responsible for the design and programming of the prototype. The ETR Institutional Review Board approved the study protocols and provided oversight. In phase one, the research team conducted formative research with potential users of the mobile app to understand the health needs of this population and how an app might support their health and to gather specific suggestions for the development and content of the app. Phase two included prototype design and development of the overall app structure and three activities (ie, a vision board, a pre-exposure prophylaxis [PrEP] education activity, and an interactive map). In phase three, a group of potential users used, rated the usability and acceptability of, and described their experiences with the prototype.

### Phase One: Formative Research

The research team conducted four focus groups and 20 in-depth qualitative interviews with transgender women between the ages of 18 and 59 years living in urban and rural areas in every region of the United States [39]. Briefly, women were recruited through partnerships with community-based organizations, fliers in community spaces and on social media, and word of mouth. Focus group participants received US $100 in compensation; interview participants received US $50. Topics included overall well-being and connectedness, transgender health, sexual health, use of internet and social media, and recommendations for a trans-specific app. Focus groups and interviews were audio-recorded and transcribed. We used the grounded theory open coding methodology and ethnographic methodologies to classify key themes [40,41].

### Phase Two: Prototype Design and Development

**Figure 2:**
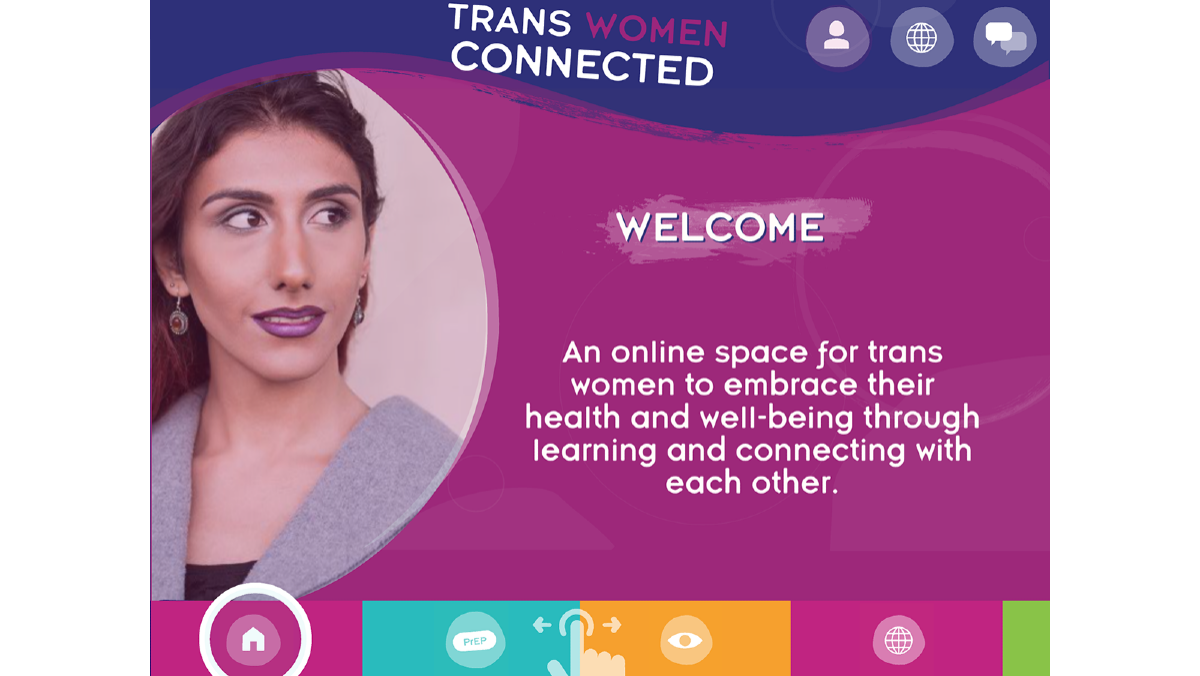
Welcome screen for the Trans Women Connected mobile app.

On the basis of the findings of the formative research, the research team developed a preliminary curriculum outline, which was discussed with and refined by the EAP and, at times, additional transgender women from their networks. The technology team then determined the optimal formats for conveying the curriculum in an engaging manner, in small self-paced segments, with sufficient levels of interactivity, and customization. The technology team next created wireframes and storyboards to mock-up the layout, workflow, and interaction of all app elements. Through an iterative process of review and feedback, the EAP guided the designer to create a final set of imagery and style guide (see Figure 2 for the overall look). Following final approval of the storyboards, the app developer created the prototype framework using Adobe Animate. Using this cross-platform development method, the app could be built for several mobile platforms, including Android and iOS, and would allow the utilization of integrated mobile device (eg, mobile phone and tablet) features such as GPS and camera.

### Phase Three: Usability Testing

#### Participants

Two community-based organizations, one in San Francisco, California and one in Portland, Oregon, recruited participants. Inclusion criteria were ages 18 to 55 years, self-identify as a transgender woman, and English speaking. All participants provided written informed consent. Participants received US $100 in compensation.

#### Procedures and Measures

Using a standard *think-aloud* protocol, the usability test administrator (TK) asked each participant to complete a series of tasks associated with typical use case scenarios, such as using the menu to locate activities and completing activities. We also objectively measured participants’ task completion success and time to completion and asked them to articulate their questions and thinking process [42,43].

Participants rated the difficulty, appearance, perceived usefulness, and educational value following each key task and after completion of all activities and completed a pre- and posttest that assessed service utilization, goal setting, social support, and HIV PrEP knowledge. We also solicited open-ended comments regarding their perceptions of the app and suggestions to improve it.

#### Analysis

We conducted descriptive analyses of the ratings and assessed changes from pre- to posttest using nonparametric tests in IBM SPSS version 24 (IBM Corp, Armonk, NY). The research team reviewed open-ended comments and identified key suggestions.

## Results

### Phase One: Formative Research

#### Themes About Transgender Health

We identified four main themes from the focus groups and interviews with 57 transgender women (45/57, 79% who were transgender women of color) related to the health of transgender women:

1. Employment and structural factors play a key role in the health of transgender women.
2. Transition-related care and mental health care are highly prioritized by transgender women. However, knowledgeable and affirming providers are difficult to find.
3. The sexual health needs of transgender women are much broader than just HIV and sexually transmitted infections.
4. Finding community and social support is challenging, but an important part of health for transgender women.

#### Conceptual Feedback About a Mobile App for Transgender Health

Our formative research found that most participants regularly used social media channels such as Facebook, Instagram, Snapchat, and various social and sexual networking apps to connect with friends, family, romantic/sexual partners, and work opportunities. However, the women also believed that the Trans Women Connected mobile app could fill a gap by providing resources specific to transgender women and an opportunity to connect to other transgender women (see Textbox 1). One Atlanta focus group participant described how this app could be a tool for “lifting the curtain and letting go of baggage.” In particular, participants valued possible app features, such as forums and direct messaging, to enable users to engage with each other and for the possibility of formal mentoring. Participants had questions and concerns about how the app would be structured, including issues of privacy and safety, and the possibility of forums and messaging being used for dating or hooking up. In general, however, participants were positive about the mobile app, and many expressed that it would fulfill an important need in the community.

**Suggestions for Trans Women Connected mobile app features and activities.**

- Give transgender women a voice in the process at every step of development
- Images and videos used in the app depict actual transgender women
- Space for trans women to connect and share information
- Optional and customizable notifications
- Customizable privacy settings to allow users to control how their information is displayed
- Information about sexual health, including sexual pleasure
- Support for healthy relationships and relationship skills
- Information to help women locate both trans-specific resources and trans friendly businesses and organizations
- Ways to block and report other users

### Phase Two: Prototype Development

**Figure 3:**
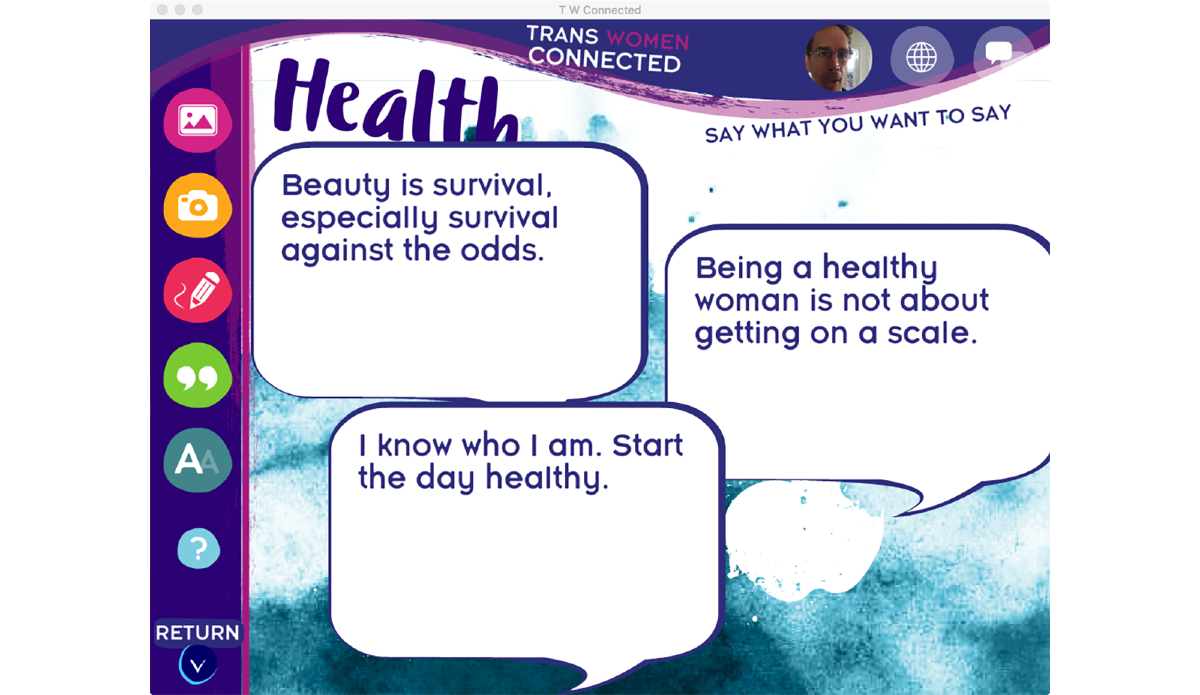
A screenshot of the Create Your Own Vision (Board) prototype activity.

Guided by the formative research and EAP, the technology team created three prototype interactive activities. The first activity, entitled Create Your Own Vision (Board), provides participants with the opportunity to imagine how they would like their lives to be in eight domains: health, living situation, school/work, sex/love, making a difference, people, free time, and spirituality (see Figure 3). This activity seeks to help transgender women situate their well-being and sexual health within the social determinants of health and structural factors that emerged as the dominant theme of the focus groups and interviews.

This interactive vision board allows participants to use their existing pictures, take pictures, create doodles/artwork, select quotes, and add their own text or other images to create a vision for each of the eight life domains. These eight collages are merged into a final scrollable collage that represents the user’s vision for their future. The activity also has the user identify a primary goal for their life today, lay out the steps needed to reach that goal, and identify who can help them execute each step. This goal setting activity provides a roadmap to reaching their goal that users can return to anytime. In addition, the vision board can be printed or shared with other app users—an option that all usability testers said they would use.

**Figure 4:**
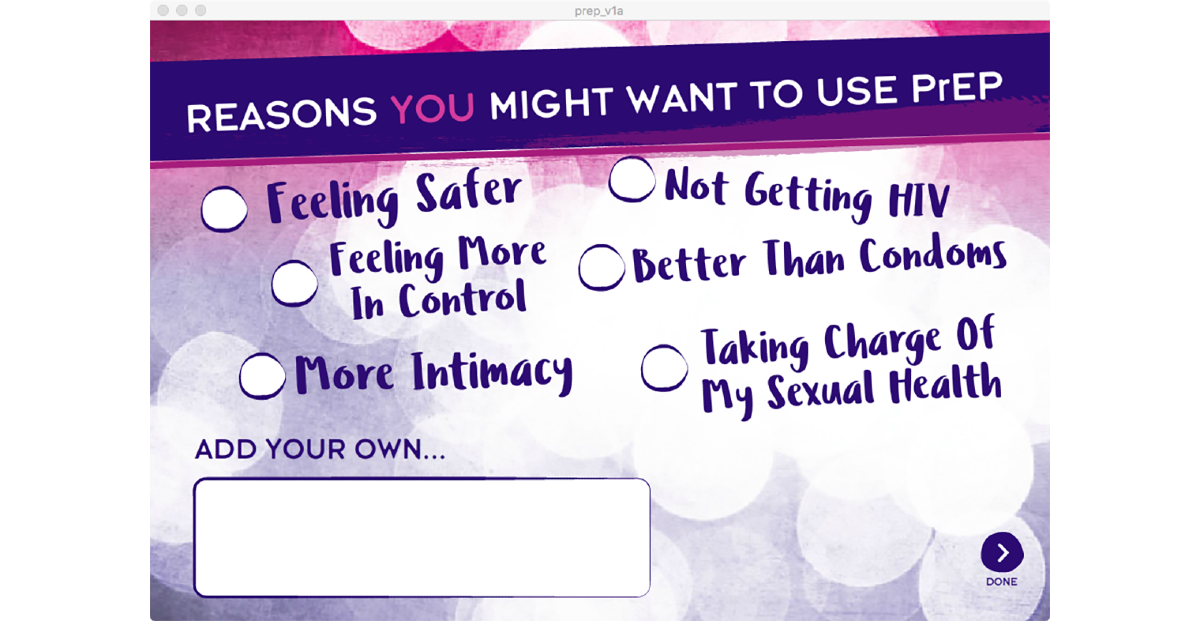
A screenshot of the Is pre-exposure prophylaxis Right for Me or My Partners? prototype activity.

**Figure 5:**
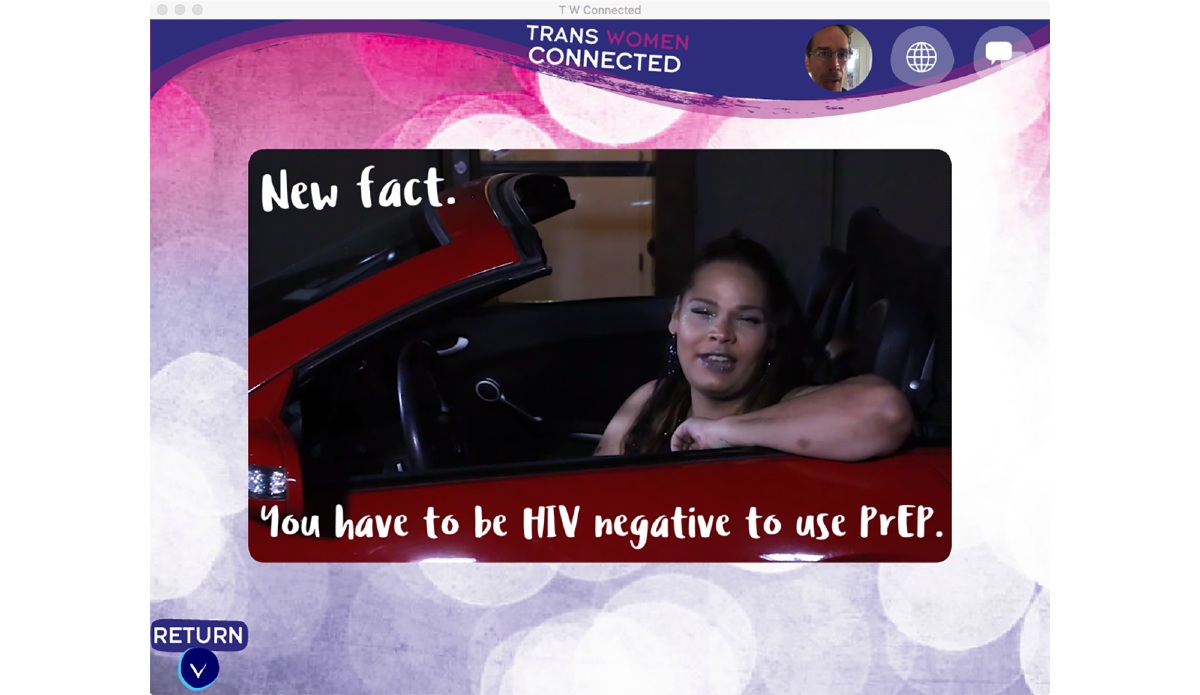
A screenshot of a video within the Is pre-exposure prophylaxis Right for Me or My Partners? prototype activity.

The second activity—Is PrEP Right for Me or My Partners?—presents a basic overview of PrEP and guides participants through a benefits and risks assessment to help them decide if PrEP might be appropriate for them or their partners (see Figure 4). The activity primarily presents information through a series of videos featuring a charismatic African American transgender woman who is getting ready for and enjoying a night out with friends, including 2 transgender women who tell personal stories about using PrEP (see Figure 5). Interactive elements were included through questions and a poll that allows the user to see what other users are thinking about PrEP and a map that identifies PrEP providers in the users’ area.

**Figure 6:**
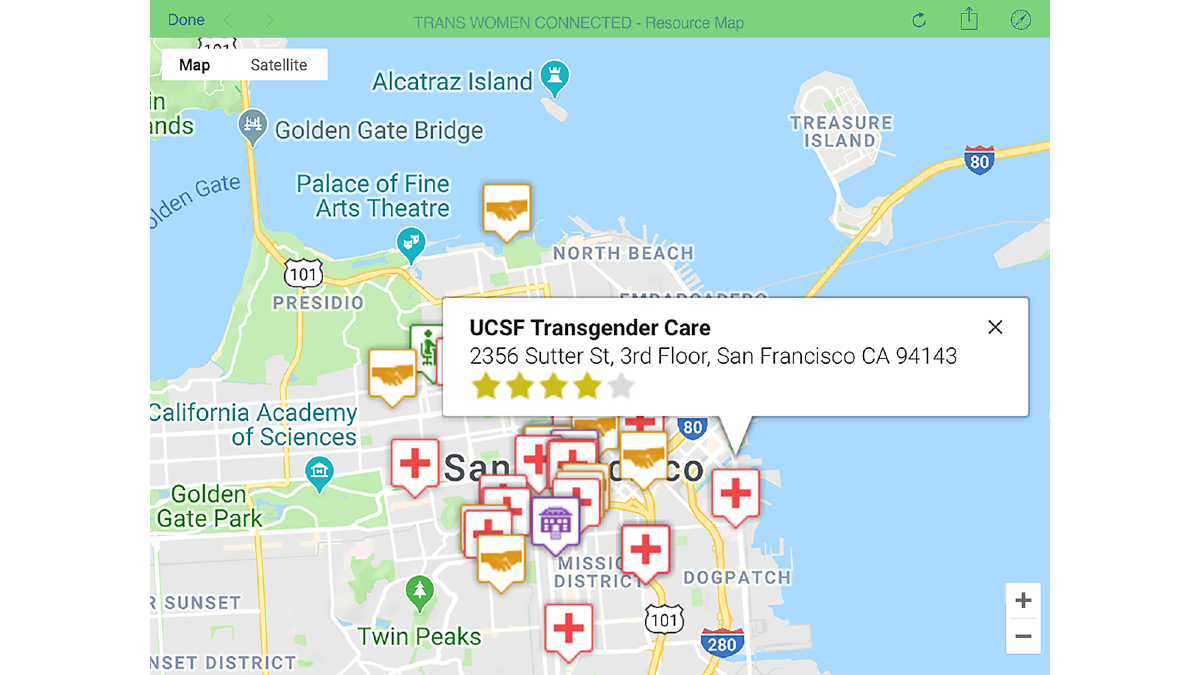
A screenshot of the Interactive Resource Map prototype activity.

Responding to the need for helping transgender women find culturally competent and welcoming services, the staff developed the third activity: Interactive Resource Map. The map displays providers/organizations in five service categories: medical, support, educational, employment, and housing/living. On the basis of GPS location, users see icons for each entry and can customize the map to view all or certain categories. When users choose an icon, they see details about the resource including name, contact information, and user ratings and reviews (see Figure 6). In addition, the map allows users to add locations to the map as they identify transgender-friendly providers in their area.

### Phase Three: Usability Testing

#### Participant Characteristics

A total of 16 racially diverse transgender women participated in the usability testing (see Table 1). Participants were aged 19 to 52 years, with an average of 34.5 years, and all were experienced with using mobile devices.

**Table 1:** Participant characteristics.

Characteristics		Values
**Location, n (%)**		
	San Francisco	11 (69)
	Portland	5 (31)
**Gender identity, n (%)**		
	Transgender woman	14 (88)
	Nonbinary	2 (13)
**Race^a^, n (%)**		
	American Indian or Alaska Native	3 (19)
	Asian	5 (31)
	Black or African American	3 (19)
	Native Hawaiian or other Pacific Islander	1 (6)
	White/Caucasian	6 (38)
	Other	3 (19)
**Ethnicity, n (%)**		
	Hispanic or Latinx	2 (13)
	Non-Hispanic or Latinx	14 (88)
Age (years), mean (SD)		34.5 (9.28)
**Experience with mobile devices, n (%)**		
	Very experienced	14 (88)
	A lot of experience	1 (6)
	Some experience	1 (6)
	Not much experience	0 (0)
	No experience	0 (0)
**Primary mode to connect with transgender women, n (%)**		
	Online	0 (0)
	In person	7 (44)
	Both online and in person	8 (50)
	Other	1 (6)
**Primary mode to meet transgender women, n (%)**		
	Online	3 (19)
	In person	9 (56)
	Both online and in person	4 (25)
	Other	0 (0)

^a^Participants could select more than one option.

#### App Usability

The usability test results demonstrated that participants liked the mobile app and found it highly usable (see Table 2). The mean rating across all usability/likability questions was 5.9 out of 7. The study benchmark was a score of 4 or better for each individual rating component.

**Table 2:** Usability and likability ratings of the Trans Women Connected mobile app.

Variable		Values, mean (SD)^a^
**Main menu**		
	Likeability of opening screen	6.25 (1.24)
	Likeability of menu system	5.66 (1.81)
**Create Your Own Vision (Board)**		
	Likeability of the appearance	6.28 (1.49)
	Likeability of the interface	6.44 (0.73)
	Likeability of the narrator	5.80 (1.14)
**Is PrEP^b^ Right for Me or My Partners?**		
	Likeability overall	5.57 (1.60)
	Likeability of women in the videos	6.14 (1.03)
**Interactive Resource Map**		
	Usefulness	6.73 (0.59)
**Overall ratings of Trans Women Connected**		
	Appearance	6.38 (1.15)
	Contents	6.39 (0.65)
	Ease of use	6.25 (1.29)
	Interest	6.08 (1.16)
	Professionally designed	5.92 (1.38)
	Enjoyability	6.25 (1.48)
	Usefulness	6.50 (1.24)
	Educational value	6.25 (0.87)
	Likelihood of using	6.58 (0.68)
	Likelihood of recommending	6.62 (1.39)

^a^7 represents the most positive or highest rating; the study benchmark was 4.

^b^PrEP: pre-exposure prophylaxis.

#### Changes in Service Utilization, Goal Setting, Social Support, and Pre-Exposure Prophylaxis Knowledge

There was a positive trend and increase across multiple measures (eg, perceptions of available social support, service utilization intention, intention to mentor other transgender women on the app, and self-efficacy in discussing PrEP; see Table 3) after using the app. Increases in self-efficacy in finding lesbian, gay, bisexual, transgender, and queer (LGBTQ)–friendly services; intention to seek online social support; and PrEP knowledge were statistically significant.

**Table 3:** Self-efficacy, intention, knowledge, goal setting, and social support: pre– and post–usability testing scores.

Variable	Usability testing scores		*P* value
	Pre	Post	
Self-efficacy in finding LGBTQ^a^-friendly services, mean (SD)	3.19 (0.75)	3.69 (0.48)	.01
Likelihood of seeking LGBTQ-friendly services, mean (SD)	3.69 (0.48)	3.56 (0.81)	.48
Intention to make medical appointment^b^, mean (SD)	3.88 (0.34)	3.88 (0.50)	>.99
Created health goals, n (%)	13 (81)	13 (81)	>.99
Considered steps to reach goal^c^, n (%)	9 (69)	9 (69)	>.99
Have social support to help reach goal, n (%)	12 (75)	14 (88)	.16
Availability of social support, mean (SD)	2.63 (1.02)	3.00 (0.97)	.71
Intention to seek online support^b^, mean (SD)	2.56 (1.09)	3.06 (1.00)	.03
Intention to seek local support services^b^, mean (SD)	3.38 (0.81)	3.50 (0.63)	.48
Helpfulness of mentoring from another trans woman, mean (SD)	4.06 (1.24)	4.19 (1.28)	.91
Intention to mentor other transgender women on app, mean (SD)	3.38 (0.89)	3.56 (0.63)	.48
Can identify friends/family for support, n (%)	15 (94)	16 (100)	>.99
PrEP^d^ knowledge, mean (SD)	2.50 (1.37)	3.56 (0.63)	.008
Intention to seek more info about PrEP^b^, mean (SD)	2.80 (1.21)	2.81 (1.22)	.58
Intention to discuss PrEP with provider^b^, mean (SD)	2.07 (0.80)	2.00 (0.97)	>.99
Intention to discuss PrEP with partner^b,e^, n (%)	6 (40)	6 (40)	>.99
Self-efficacy in discussing PrEP, mean (SD)	3.00 (1.13)	3.25 (0.86)	.16
Connection to other transgender women, mean (SD)	6.44 (2.60)	6.63 (2.33)	.78
Satisfaction with connection to other transgender women, mean (SD)	5.00 (1.67)	5.19 (1.83)	.26
Level of social support from other transgender women, mean (SD)	5.19 (1.87)	5.21 (1.81)	.72

^a^LGBTQ: lesbian, gay, bisexual, transgender, and queer.

^b^In the next 30 days.

^c^Out of 13 participants.

^d^PrEP: pre-exposure prophylaxis.

^e^Out of 15 participants.

#### Qualitative Findings From Usability Testing

Usability testing participants also provided a number of qualitative comments about their experience using the prototype and suggestions for how it might be improved (see Textbox 2). Overall, this qualitative feedback was positive. As one participant told us, “Helping trans people navigate their health is a win.” As in the quantitative ratings, women expressed that they felt the app was useful and attractive. The welcome screen was described as being friendly and approachable, and many participants listed the esthetics of the app as one of their favorite parts. The women liked all three activities, with the map being a particular favorite. The potential for interactivity and connection with other transgender women was described as another attractive feature. The suggestions provided ranged from broad to very specific; a list of some of the most common or useful suggestions can be seen in Textbox 2.

**Suggestions to improve the Trans Women Connected mobile app prototype.**

General

- More information or instructions on welcome screen and main menu
- Tidier appearance/colors
- More photos with a variety of transgender women (more representative of the community)
- Options for text instead of audio; subtitles for all audio
- Change or remove audio when returning to a screen
- More black trans women developers
- Provide suggestions for donating or volunteering

Create Your Own Vision (Board)

- Improve instructions and make navigation clearer
- Ability to draw directly on vision board
- Ability to add songs, gifs, or other “moving” content
- Option to turn off narration
- Option to share on social media

Is PrEP Right for Me or My Partners?

- Option to pause videos
- Shorter videos
- Option to view more scientific information
- Clarify some information

Interactive Resource Map

- Add a search bar and list of locations
- Ability to filter user-added locations
- Filter or information on Americans with Disabilities Act-accessible locations
- Add other types of resources, such as gender-neutral restrooms
- Add a filter or new category for free activities

## Discussion

### Principal Findings

We conducted a 3-phase process to develop a prototype HIV prevention mobile app for and with transgender women. In the first phase, we completed formative research, which indicated that HIV prevention for transgender women must address the larger context of women’s lives. In the second phase, we developed and refined the overall app framework and three prototype activities based on feedback from the EAP. In the third phase, we conducted usability testing. Participants rated the app highly on usability and likeability. There was a significant increase in self-efficacy in finding LGBTQ-friendly services, intention to seek online social support, and PrEP knowledge. Overall, the participants were supportive of the app, with many saying they would use it regularly with no additional incentive beyond the information and opportunity to connect with other transgender women.

### Strengths of the Approach

There are a number of strengths of the Trans Women Connected prototype mobile app and research process. The 3-phase development process allowed us to collect formative data about the health priorities of transgender women and incorporate this information into the app prototype. We developed these activities because they addressed issues or requests observed in the formative research. This approach, which is considerably more extensive than the typical app development approach, helps to ensure that the selected prototype activities were addressing real needs of the community. The extensive formative research and the rigor of the analysis in this project, when combined with an iterative usability testing approach, are unique among mobile app developers.

Although researchers have called for digital health interventions to (1) be grounded in behavioral theory [44,45], (2) utilize an in-depth qualitative understanding of the intended population [46], and (3) be iteratively developed with multiple stages of user feedback [47,48], the process has been rarely implemented consistently, with variations in the development process between academic researchers and technology industry developers. Most mHealth interventions designed by those within the industry do not yet incorporate theory-based strategies known to drive changes in health behaviors or undergo systematic testing to demonstrate their effectiveness [45,49,50]; however, they typically employ a rapid iterative development process of the technology. In contrast, interventions developed within an academic environment are more often grounded in behavioral theory [51], but researcher-driven mobile interventions often do not benefit from rapid iterative prototyping and standardized usability testing with iterative feedback [52].

The development of this app integrated the strategies used in both academia and the industry and, in some instances, used methodologies that were more rigorous. For example, rather than use a local convenience sample for focus groups, as is typical of most software and app developers, the project conducted focus groups with transgender women in four regions and interviews with transgender women throughout the United States, with key transgender community stakeholders actively assisting in recruitment activities. By using a community-driven, grounded theory approach, the research team was able to identify and delve deeply into themes that would not have been detected through simply examining focus group transcripts from convenience samples.

Furthermore, input from transgender women was integrated throughout the prototype development in coordination with the EAP and women from their networks. These iterative feedback loops began earlier than typically in app development and included gathering feedback on colors and image representations of transgender women and several other functional and aesthetic elements of the app before moving on to feedback on the app interface and activity content, function, and appearance. The inclusion of members of the LGBTQ community as part of the development process extended beyond formative research into voice talent/narration, video development, and design, with the designer being an LGBTQ-identified woman of color, the narrators being transgender women of color, and all video participants being transgender women. These multiple forms of community engagement not only resulted in a highly tailored app but also further provide a foundation for dissemination and active utilization once the Trans Women Connected mobile app is finalized. As suggested by other researchers, community engagement increases the likelihood the knowledge gained and the intervention, in this case an app, will benefit the community [53-55]. We recognize and appreciate the importance of the community’s priorities and solutions, which enhances the relevance and use of data and quality and validity of research [56-58].

Additional strengths of the usability testing were the multiple types of data and an evaluation of impact. We gathered both quantitative and qualitative data about the experience of using the prototype mobile app; the quantitative data allowed us to verify that we were meeting numeric targets for usability and acceptability (as is typical for usability testing), whereas the qualitative data gave participants the opportunity to provide more in-depth feedback and suggestions. However, measuring the impact of the prototype on knowledge, attitudes, and intentions during the development process rather than when the app is completed goes considerably beyond standard usability testing, which is typically limited only to identifying problems within the user experience [59]. This approach helps determine whether the learning and engagement activities are performing as intended before building out the entire app.

Finally, a central strength of this mobile app is the emphasis on addressing HIV holistically using a strengths-based perspective. This approach recognizes the resilience of the transgender community and the social and structural barriers that transgender women face [60]. Developing an effective intervention requires a comprehensive approach that intentionally targets community strengths and challenges [60-63]. Our formative research confirmed that attention to the larger context of trans women’s lives was key to HIV prevention [14], [61,64,65], and this understanding guided our development process.

### Limitations

A few limitations of our study need to be considered. The usability testing sample was limited to 16 participants in two different cities in the West Coast. Research has determined that 7 to 10 usability participants will uncover 80% to 90% of usability problems [66,67], and we were able to test the mobile app with a diverse group of 16 participants. This sample allowed the examination of some of the different experiences and identities of transgender women. We likely identified functional problems with the app and the app was well received as usable and acceptable; however, it is possible that more geographically diverse participants or participants from rural areas would have qualitatively experienced the app differently.

The inclusion of pre- and posttest knowledge and intention measures during a usability test is unusual and presents some limitations. Although significant increases in knowledge and intent were found on some variables, the sample size, although adequate for usability testing, is small for such testing and again may not represent the effect that would have been found with a more diverse or larger sample. However, the results are promising given that there were positive changes in key measures after viewing only three prototype activities, and this would seem to bode well for the efficacy of the complete mobile program. For several measures, there was no significant increase between pre- and posttest scores; we may have observed a ceiling effect. Given the short duration of app use, we were unable to measure behavior change. However, we measured antecedents of behavior change according to behavior change theories, and in the full trial when participants have a longer period to use the mobile app, we expect to observe increases in these antecedents and more protective behaviors.

A final limitation of the project was that all members of the research team identify as cisgender. Although the input of transgender women was included throughout the development process, no transgender individuals participated directly in the interpretation and analysis of data. As a result, there may be aspects of the data that were missed or misinterpreted because of the absence of this perspective.

### Next Steps

Given the positive results from the prototype development and usability testing, our next steps are to complete the development of the mobile app, including the storyboards and curriculum/content for the remaining activities, and conduct a cluster randomized controlled trial to assess the effectiveness of the Trans Women Connected mobile app. The expansion of the app to include additional activities will be based on a theoretical framework of gender affirmation, resilience, and cognitive behavioral theory and will be implemented using an agile development process with iterative testing. Through the entire process, we will continue to work with an expanded expert and community advisory group of transgender women who will provide feedback on the app; they will review materials to ensure the content reflects their experiences and those of their community. The project team, in collaboration with the expert advisors, will identify the most essential topics, which will then be reviewed and revised through an iterative process of refinement. A newly formed community advisory board, consisting of 10 geographically and racially diverse transgender women, will provide feedback on the activity plans and storyboards. The project team will then revise the documents, with the process continuing until the content is approved by the community advisors. In addition, the community advisory board will test and review completed interactive activities throughout the project.

### Conclusions

Strong formative research, followed by an iterative review from members of the intended audience, resulted in a usable and acceptable mobile app that has been well received by transgender women. Usability testing findings provided important insights toward refining and the further development of the Trans Women Connected mobile app. The results of this study suggest that an mHealth intervention can address critical structural factors that shape transgender women’s lives and support positive changes in the knowledge and attitudes about social connection and health access that are antecedents to increasing protective and health promotion behaviors.

## Abbreviations

CDC: Centers for Disease Control and Prevention
EAP: expert advisory panel
eHealth: electronic health
LGBTQ: lesbian, gay, bisexual, transgender, and queer
mHealth: mobile health
PrEP: pre-exposure prophylaxis
